# Turnover and flow of the cell membrane for cell migration

**DOI:** 10.1038/s41598-017-13438-5

**Published:** 2017-10-11

**Authors:** Masahito Tanaka, Takeomi Kikuchi, Hiroyuki Uno, Keisuke Okita, Toshiko Kitanishi-Yumura, Shigehiko Yumura

**Affiliations:** Department of Functional Molecular Biology, Graduate School of Medicine, Yamaguchi University, Yamaguchi, 753-8512 Japan

## Abstract

The role of cell membrane dynamics in cell migration is unclear. To examine whether total cell surface area changes are required for cell migration, *Dictyostelium* cells were flattened by agar-overlay. Scanning electron microscopy demonstrated that flattened migrating cells have no membrane reservoirs such as projections and membrane folds. Similarly, optical sectioning fluorescence microscopy showed that the cell surface area does not change during migration. Interestingly, staining of the cell membrane with a fluorescent lipid analogue demonstrated that the turnover rate of cell membrane is closely related to the cell migration velocity. Next, to clarify the mechanism of cell membrane circulation, local photobleaching was separately performed on the dorsal and ventral cell membranes of rapidly moving cells. The bleached zones on both sides moved rearward relative to the cell. Thus, the cell membrane moves in a fountain-like fashion, accompanied by a high membrane turnover rate and actively contributing to cell migration.

## Introduction

**Figure 1:**
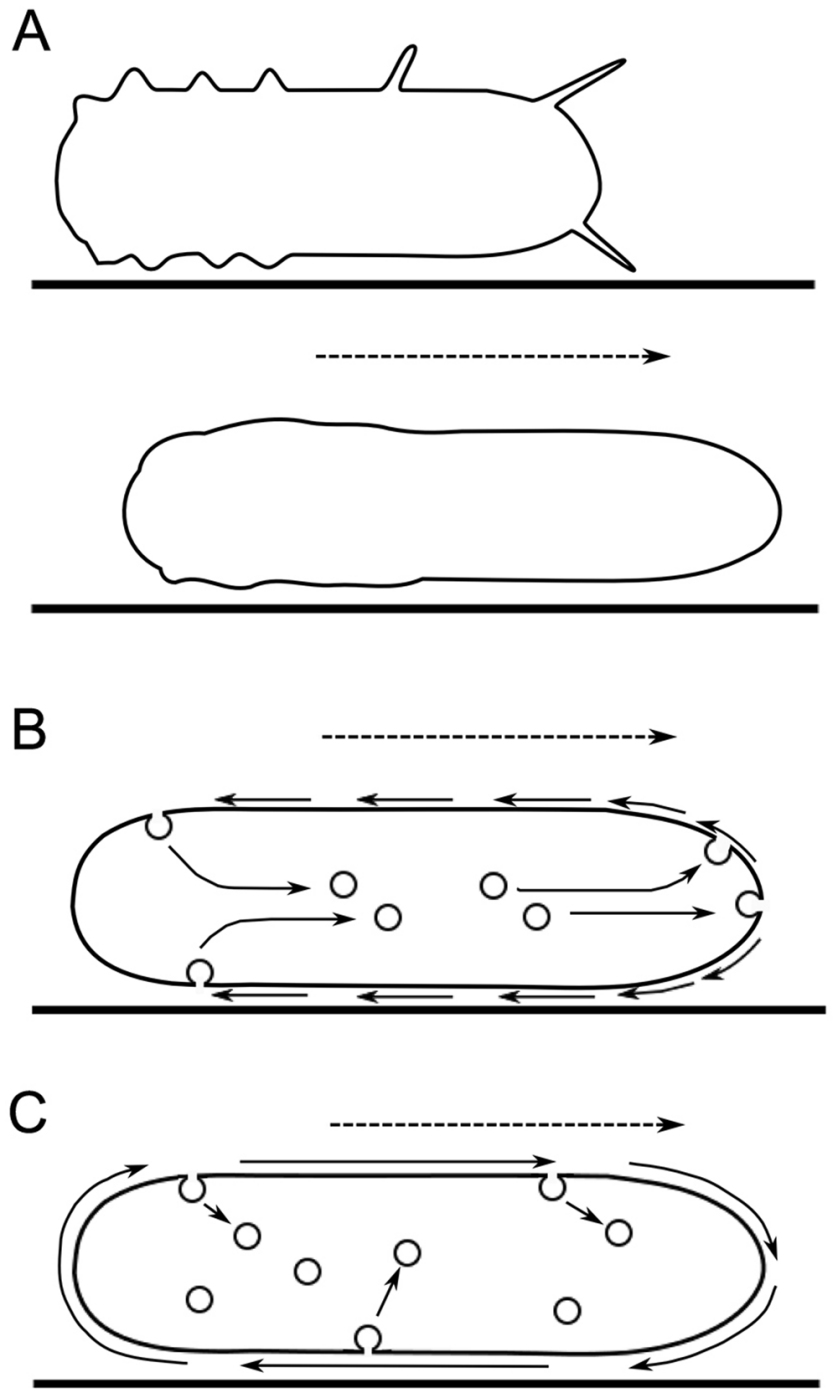
Three models for the behavior of the cell membrane during cell migration. In a membrane unfolding model (**A**), the cell changes its shape during migration by alternating between folding (upper panel in **A**) and unfolding (lower panel in A) the cell membrane. The folded surface appears as projections and wrinkles on the cell surface and is utilized as a membrane reservoir. In the fountain flow model (**B**), both the dorsal and the ventral membrane flow toward the rear of a migrating cell; membrane precursor vesicles fuse with the anterior cell membrane to supply membrane (exocytosis), and membrane is taken up at the rear (endocytosis). In the caterpillar flow model (**C**), the cell membrane moves circularly in the order of the ventral, anterior, dorsal, and rear regions. In this case, the cell membrane may turn over everywhere. The dotted arrows show the direction of cell migration. The solid arrows indicate the direction of trafficking and membrane flow.

Cell migration plays important roles in many cellular processes, such as morphogenesis, immune responses, and wound healing. The cytoskeleton has been well established to contribute to cell migration. Cells migrate by extending anterior pseudopods via a pushing force generated by the assembly of actin filaments and retracting their rear by a contractile force of actomyosin^1,2^. In this context, the cell membrane at the anterior must be enlarged to extend the pseudopods. However, the cell membrane can physically stretch at most 2–3%^3^. The expansion of the cell surface (cell membrane) can be explained either by the utilization of a folded membrane surface as a reservoir or by the exocytosis of internal vesicles, which remains controversial. In the first model (Fig. 1A), cell surface projections and folds are lost or gained coincident with cell surface expansion or shrinkage during cell shape changes, in a manner reminiscent of the bellows of an accordion. This idea (the membrane unfolding model) came originally from studies of free-living amoebae^4^ and has been supported in many species of cells by scanning electron microscopy and recent live cell imaging^5–7^. Chen proposed ‘retraction induced spreading hypothesis’, from the observations that the retraction of the trailing edge resulting in the folding of cell surface proceeds spreading at the leading edge of fibroblasts^8^. On the other hand, in support of the latter model (Fig. 1B and C), many pieces of evidence have accumulated to show that exocytosis and endocytosis from the internal membrane stores contribute to cell migration^9,10^.

The cell membrane is always refreshed by membrane insertion via the exocytic fusion of membrane precursor vesicles and membrane removal via endocytic uptake. In slowly moving cells such as fibroblasts, the internalized membrane vesicles are returned to the leading edge, which should help with extension for forward cell migration. The membrane area taken up each minute is about the same as that required to extend the front of the cell^11^. However, a more rapid supply of new cell membrane is required for more rapidly migrating cells, such as leukocytes and *Dictyostelium* cells.

The time required for exchanging the total cell membrane has been examined in *Dictyostelium* cells. Internalization of isotope-labeled surface proteins indicated a time of 45 min for total cell membrane exchange^12^. Internalization of the cell membrane stained with a fluorescent lipid analogue (FM1-43) revealed a 4–10 min turnover time in vegetative cells, which may be reasonable to explain the contribution of cell membrane turnover to cell migration^13^. However, these authors examined cells in a vegetative stage, where the cells actively eat the external nutrient medium. In addition, they examined the measurements in a suspension condition, where the cells could not migrate. Thus, it is difficult to discuss the direct relationship between cell membrane turnover and cell migration.

If new cell membrane is supplied through the cytoplasm and old membrane is taken up from the cell surface as the cell migrates, how does the cell membrane behave? There are two models for the circulation of the cell membrane during cell migration: the fountain flow model and the caterpillar flow model. In the fountain flow model (Fig. 1B), both the dorsal and ventral membranes flow toward the rear of migrating cells; membrane precursor vesicles are fused to the anterior cell membrane to supply new membrane lipids, and at the rear, membrane is removed by internalization. The fountain flow model was originally called the ‘retrograde flow model’^14^, but this is sometime confused with actin retrograde flow^15,16^. Thus, here we will use the term ‘fountain flow’. In the caterpillar flow model (Fig. 1C), the cell membrane moves circularly in the order of the ventral, anterior, dorsal, and rear regions. In this case, the cell membrane may turn over everywhere.

In previous experiments to examine the above models, after the cell membrane of polymorphonuclear leukocytes was stained with a fluorescent lipid analog, a small linear region was photobleached as a marker. The bleached marker moved forward with the same velocity as the cell advanced. This result refutes the fountain flow model^17^. However, due to a technical limitation at that time, the photobleaching was simultaneously performed on both the dorsal and ventral cell membrane in single cells, and the individual movements could not be distinguished.

In the present study, we first examined whether the cell surface area changes during cell migration using improved experimental approaches, and we could not find any changes in the total cell surface area during cell migration. Next, we found a direct relationship between the rate of cell membrane internalization and the velocity of cell migration. Furthermore, we demonstrate that the cell membrane flows rearward, as the fountain flow model assumes, and provide essential information regarding the mechanism of cell migration.

## Results

### The total area of the cell surface is constant during cell migration

**Figure 2:**
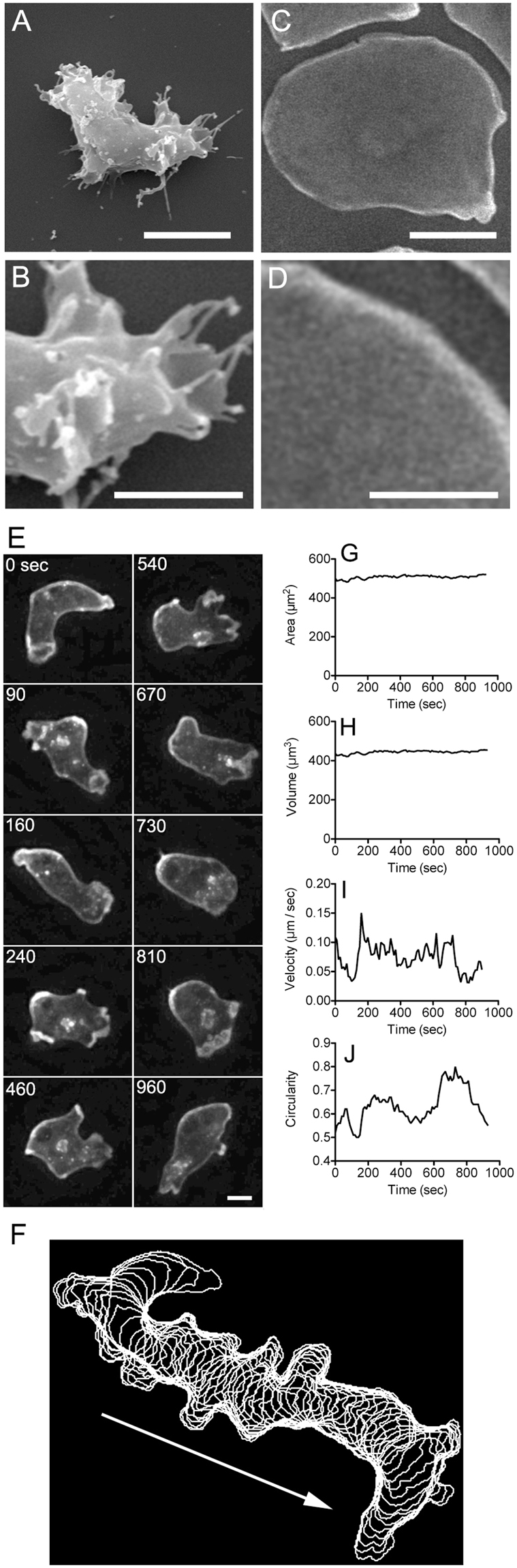
The total cell surface area does not change during cell migration. Typical scanning electron micrographs of *Dictyostelium* cells without (**A** and **B**) and with (**C** and **D**) agar-overlay. In the case of agar-overlay, the cells were observed after the agar sheet was removed. Panels B and D show enlarged images of panels A and C. The cell without the agar-overlay had many wrinkles, as well as small and long projections, on its surface. In contrast, the cells pressed with agar-overlay were flattened and there was no sign of wrinkles or projections on the cell surface. Bars in panels A and C, 5 µm; Bars in panels B and D, 2.5 µm. (**E**) Representative time-lapse 3D-images from z-sectioning stacks of a migrating pre-aggregation cell expressing GFP-ABD under agar-overlay. Each cell image is cropped to identically sized squares, and thus does not show the actual displacement of the cell (see also Supplementary Video S1). Bar, 5 µm. (**F**) Time-lapse image stacks obtained from the cell boundary of panel E at 20 sec intervals. Arrow indicates the direction of cell migration. The surface area (**G**), cell volume (**H**), cell velocity (**I**), and circularity (**J**) of the cell shown in panel E are plotted over time. Note that the cell surface area and cell volume did not change although the cell shape changed vigorously. We confirmed this result with independent experiments using 20 different migrating cells (see Supplementary Table S1). There was no correlation between the velocity and cell circularity (the Pearson’s correlation coefficient was 0.026 ± 0.332, n = 20). The mean cell velocity did not change when GFP-ABD was expressed or when cells were labeled with Cell Mask Orange, compared with untreated control cells (3.38 ± 0.81, 3.30 ± 0.68 and 3.57 ± 0.83 μm/min, respectively). Data are presented as mean ± SD and analyzed by one way ANOVA with Tukey’s multiple comparison test. (n = 20 each).

Migrating *Dictyostelium* cells always change their shape by extending pseudopods and retracting their rear. Previous experiments have shown substantial dynamic changes of up to 20–30% in cell surface area during migration in *Dictyostelium* cells^18^. However, we previously showed using scanning electron microscopy (SEM) that there are many projections and wrinkles on the cell surface^19^, which may complicate the accurate measurement of the cell surface area. Here, to lessen small projections and surface wrinkles, the cells were pressed with an agar block to expand the cell membrane. Without agar-overlay, the cells were 7–9 µm in height, and agar-overlaid cells became flat, with a thickness of about 2 µm. Even under this condition, the cells could migrate extending large pseudopods. Blebs were also observed only during the initial pressuring with the agar overlay^20^. The cells under agar-overlay did not extend thin extensions such as filopods and microvilli as far as we observed under a light microscope. To confirm this, the cells were observed using scanning electron microscopy. An agar block was removed to observe the cell surface after fixation (see Materials and Methods). Whereas the cells without agar-overlay had many wrinkles and projections on their surface (Fig. 2A and B), the cells under agar-overlay had flattened shapes and no signs of wrinkles and projections on the cell surface (Fig. 2C and D). There could be minute wrinkles or folds in the cell membrane under the resolution of SEM. However, we could not find any such minute wrinkles or folds under transmission electron microscopy, which has higher resolutions than SEM (Supplementary Fig. S1).

To examine the total volume and total surface area of migrating cells, cells expressing GFP-ABD, a marker of actin filaments, were pressed with agar-overlay, and a z-series of fluorescence images was acquired by optical sectioning fluorescence microscopy, processed using deconvolution to remove out-of-focus signals, and then reconstituted as 3D images (Fig. 2E). In this figure, each cell image is cropped to identically sized squares, and thus does not show the actual displacement of the cell. Figure 2F shows time-lapse image stacks obtained from the cell boundary of Fig. 2E at 20 sec intervals, indicating that the cells can migrate under agar-overlay (see also Supplementary Video S1). Figure 2G–J show a typical time course of the cell surface area, cell volume, instantaneous velocity and cell circularity, respectively. As the value of cell circularity approaches 1, the cell shape becomes round (see Materials and Methods). Whereas the cell changed its morphology with fluctuating velocity (Fig. 2I and J), there was almost no change in the surface area and volume (Fig. 2G and H). We confirmed this result with independent measurements of 20 different migrating cells. The maximal changes in relative surface area and volume were 5.01 ± 1.62% and 5.17 ± 1.79%, respectively (Supplementary Table S1). From these experiments, we concluded that cells are able to deform without any cell surface reservoirs such as projections and wrinkles.

### Uptake of cell membrane during cell migration

Aguado-Velasco and Bretscher (1999) showed that the uptake of cell membrane equivalent to one cell surface area takes 4–10 min in vegetative cells, which may reasonably explain the contribution of cell membrane turnover to cell migration. However, vegetative cells are very active in internalizing the nutrient medium by macropinocytosis. In addition, the group performed the measurements in a suspension culture condition, where the cells could not migrate. In the present study, we directly investigated membrane internalization in individual migrating cells in a non-vegetative stage (pre-aggregation and aggregation-competent stages), when the endocytic and pinocytic activities are greatly reduced^21^.

**Figure 3:**
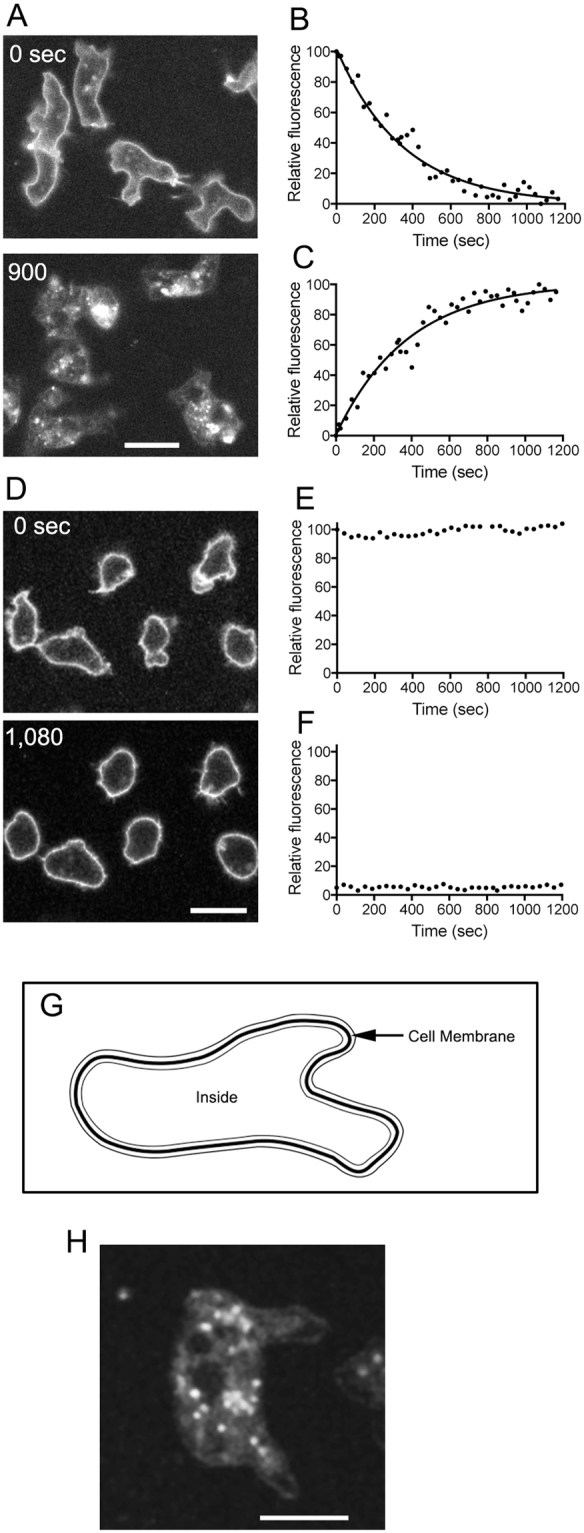
Uptake of cell membrane during cell migration. *Dictyostelium* cells were stained with CellMask Orange, a fluorescent lipid analogue. Within 1 min after adding the probe, the cell membrane was stained uniformly, as observed by confocal microscopy (upper panel in A). The fluorescence was internalized as the cell migrated, suggesting that cell membrane is taken up inside the cell (lower panel in A). As shown in panel G, the integrated fluorescence intensity of the cell membrane and inside the cells were calculated (see details in Materials and Methods). Graphs **B** and **C** show typical time courses of the fluorescence intensities of the cell membrane and inside the cells, respectively. The integrated fluorescence at the cell membrane decreased with a half-life of 324 ± 139 s (n = 23 cells). The integrated fluorescence inside the cells increased in a manner inversely correlated with the fluorescence at the cell membrane. Therefore, half of the total lipids in the cell membrane are internalized and newly exchanged within approximately 5 min. (**D**) Cells were stained with CellMask Orange in the presence of sodium azide (upper panel in D). The fluorescence of cell surface did not show any change and the fluorescence intensity inside the cells did not increase (lower panel in D). Graphs **E** and **F** show typical time courses of the fluorescence intensities of the cell membrane and inside the cells, respectively. (**H**) After stained with CellMask Orange, the cells were overlaid with an agar block. Note that the probe was already internalized and found in many intracellular vesicles about 5 min after the agar-overly. Bar, 10 µm.

Migrating *Dictyostelium* cells (pre-aggregation stage) on a coverslip were stained with CellMask Orange, a fluorescent lipid analogue, and observed by confocal microscopy. Once this probe is inserted into the cell membrane, it emits an orange colored fluorescence under illumination by green light. The cell membrane was stained uniformly within 1 min after applying the probe (upper panel of Fig. 3A). After removing the free probe in the buffer, fluorescent images were acquired over time. The fluorescence was internalized gradually as the cell migrated, and finally, many fluorescent vesicles were observed inside the cells, which is presumably a result of endocytosed membrane (bottom panel of Fig. 3A).

The integrated fluorescence intensities of the cell membrane and the cell interior were measured as described in the Methods section (Fig. 3G). Figure 3B and C shows the time courses of the fluorescence intensities of the cell membrane and the cell interior, respectively. The fluorescence at the cell membrane decreased, with a half-life of 324 ± 139 s (n = 23). The fluorescence inside the cells increased in a fashion opposite to that of the cell membrane. Thus, half of the total lipids of the cell membrane were internalized and newly exchanged within approximately 5 min.

In the presence of sodium azide, an uncoupler of oxidative phosphorylation, the fluorescence intensity inside the cells did not increase and the surface staining did not change (Fig. 3D). Figure 3E and F show the time courses of the fluorescence intensities of the cell membrane and the cell interior, respectively, which suggest that the probe does not cross the cell membrane. In addition, the internalization of cell membrane is due to the endocytosis, which is energy-dependent^13^.

We could not examine the time course of cell membrane internalization under agar-overlay, because the agar-overlay took more time than without agar-overlay. After this preparation, we found that the probe was already internalized into small vesicles in the cytoplasm (Fig. 3H). In any case, it is clear that the cells rapidly internalize cell membrane.

**Figure 4:**
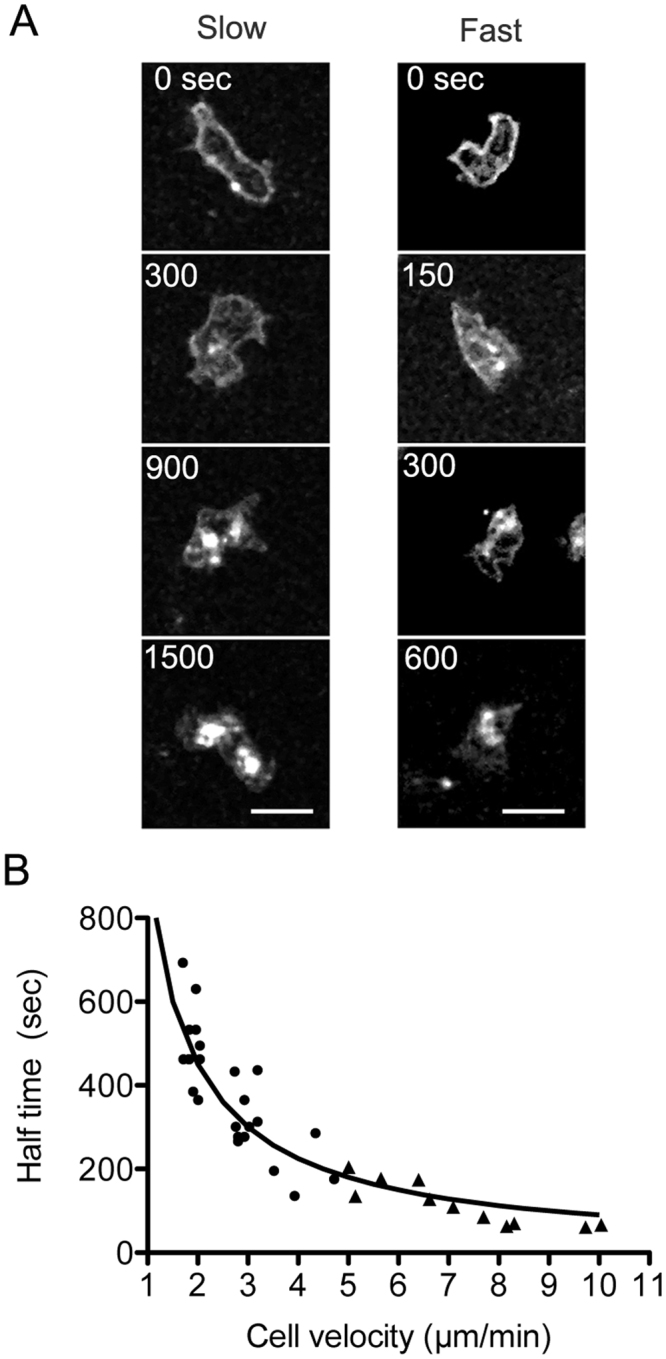
Uptake rate of the cell membrane is correlated with cell velocity. (**A**) Two typical images of the time course of cell membrane internalization (slowly- and fast-migrating cells). Each cell image is cropped to identically sized squares, and thus does not show the actual displacement of the cell. Note that the staining with CellMask Orange remained for a longer period in the cell membrane of slowly-moving cells than in that of fast-migrating cells. Bars, 5 µm. (**B**) The relationship between the half-life of cell membrane internalization and the cell velocity. The cell velocity was calculated from time-lapse images using imageJ software. Note that faster-migrating cells internalize the cell membrane more rapidly than slower cells. Pre-aggregation cells (circles) and aggregation-competent cells (triangles) are indicated.

We frequently observed that the staining with CellMask Orange remained for a longer period of time in the cell membrane of slowly-migrating cells than in that of fast-migrating cells (Fig. 4A). The uptake of the lipid could be related to the velocity of cell migration. Thus, we examined the cell membrane internalization in aggregation-competent cells, which migrate much faster than pre-aggregation cells. An average half-life of the internalization was 106 ± 45 s (n = 11), which is much faster than that of pre-aggregation cells. Figure 4B shows the relationship between the half-life of membrane internalization and the cell velocity of pre-aggregation cells (circles) and aggregation-competent cells (triangles), indicating that faster migrating cells internalized their membrane lipids more rapidly. In the fastest cell, the half-life was approximately 60 s.

If a migrating cell should refresh its total cell membrane relative to the advance of its total body length (20 µm), the half-life should be 60 s when the cell migrates with a velocity of 10 µm/min, which is consistent with the actual value observed.

### Lipid movement by lateral diffusion in the cell membrane independently of the cytoskeleton

There are two models for the circulation of the cell membrane during cell migration: the fountain flow model and the caterpillar flow model (Fig. 1). To clarify which model is suitable, photobleaching experiments were performed separately on the dorsal and ventral cell membranes. If both bleached zones moved rearward, the membrane moved in a fashion consistent with the fountain flow model (Fig. 1B). If the bleached zone in the dorsal cell membrane moved forward and that in the ventral cell membrane moved rearward, the membrane moved in a fashion consistent with the caterpillar flow model (Fig. 1C).

**Figure 5:**
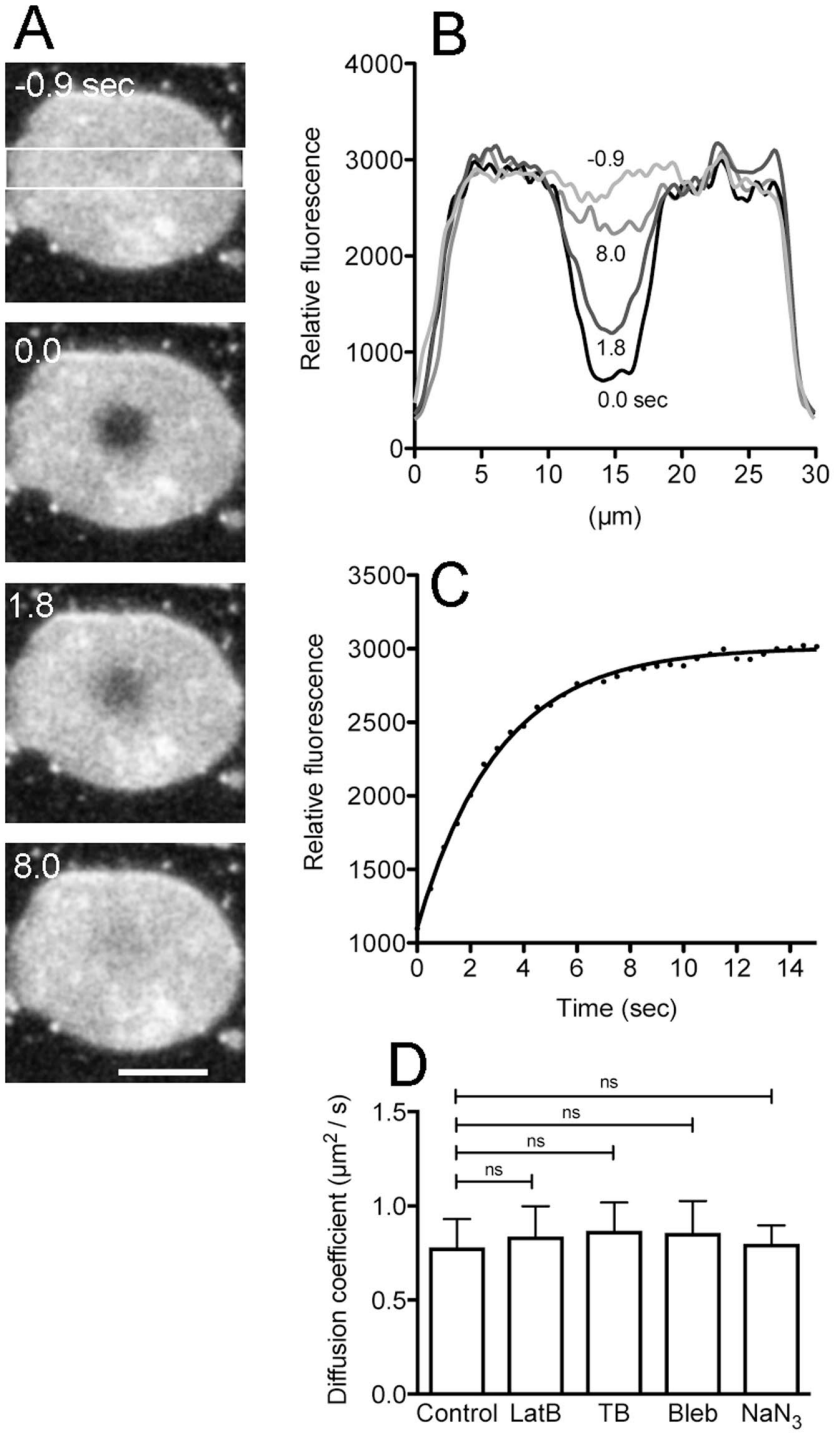
Lateral diffusion of lipids is not affected by cytoskeletons. The cell membrane was stained with Polaric, a similar fluorescent lipid analogue to CellMask Orange. (**A**) Typical fluorescence images when a small region of the cell membrane was photobleached (0 sec). (**B**) The fluorescence intensity profiles in the white rectangle in panel A at each time (−0.9, 0.0, 1.8, and 8.0 sec). Note that the fluorescence recovered by a simple lateral diffusion of the lipids. (**C**) A time course of fluorescence recovery in the bleached circles. (**D**) A comparison of the diffusion coefficient in the presence of inhibitors (latrunculin B, thiabendazole, blebbistatin, and sodium azide), indicating that all these inhibitors do not affect the lateral diffusion of the membrane lipids (n = 20 each). Data are presented as mean ± SD and analyzed by one way ANOVA with Tukey’s multiple comparison test. ns, not significant. Bar, 10 µm.

Before these experiments, the lateral diffusion of lipids in the cell membrane was characterized. The cell membranes were stained with Polaric, a fluorescent lipid analogue similar to CellMask Orange, and a small part of the cell membrane was photobleached (0 sec in Fig. 5A). The fluorescence of CellMask Orange could not be bleached by the maximum power of our HeNe laser, and thus we changed the fluorescent lipids to Polaric, the fluorescence of which could be easily bleached by our argon laser. Polaric also showed a similar kinetics of internalization of cell membrane to CellMask Orange (data not shown). After bleaching, the bleached area gradually closed from the periphery, indicating the fluorescent recovery within this short period could not be caused by the supply of cell membrane via exocytosis, but by a simple lateral diffusion of lipids (Fig. 5B). Figure 5C shows a time course of fluorescence recovery in the bleached area. The half-life of recovery was 2.00 ± 0.47 s (n = 26), and the diffusion coefficient was 0.78 ± 0.15 µm^2^/s, which is comparable to previous measurements of animal culture cells^22^.

Next, to examine whether the lateral movement of lipids is affected by actin filaments, microtubules, or myosin II, the cell membranes were photobleached in the presence of inhibitors, latrunculin B, thiabendazole, or blebbistatin. In addition, sodium azide was used as a positive control. Figure 5D shows that all these inhibitors did not affect the diffusion coefficients, which were calculated from the half-life of recovery. Therefore, the lipids freely moved in the cell membrane by simple lateral diffusion independently of the action of cytoskeleton.

### Circulation of the cell membrane during cell migration

To distinguish between the above two membrane flow models using photobleaching experiments, the cells must advance rapidly enough to detect the movement of bleached marker zones before their fluorescence is recovered by diffusion. If the marker is 5 µm in width, then its lifetime will be approximately 3 sec. The cell should advance at least 0.5 µm for us to easily detect the advance of the membrane within this time (i.e., 10 µm/min in cell velocity). *Dictyostelium* is a suitable candidate for studying migrating cells with such a high velocity.

**Figure 6:**
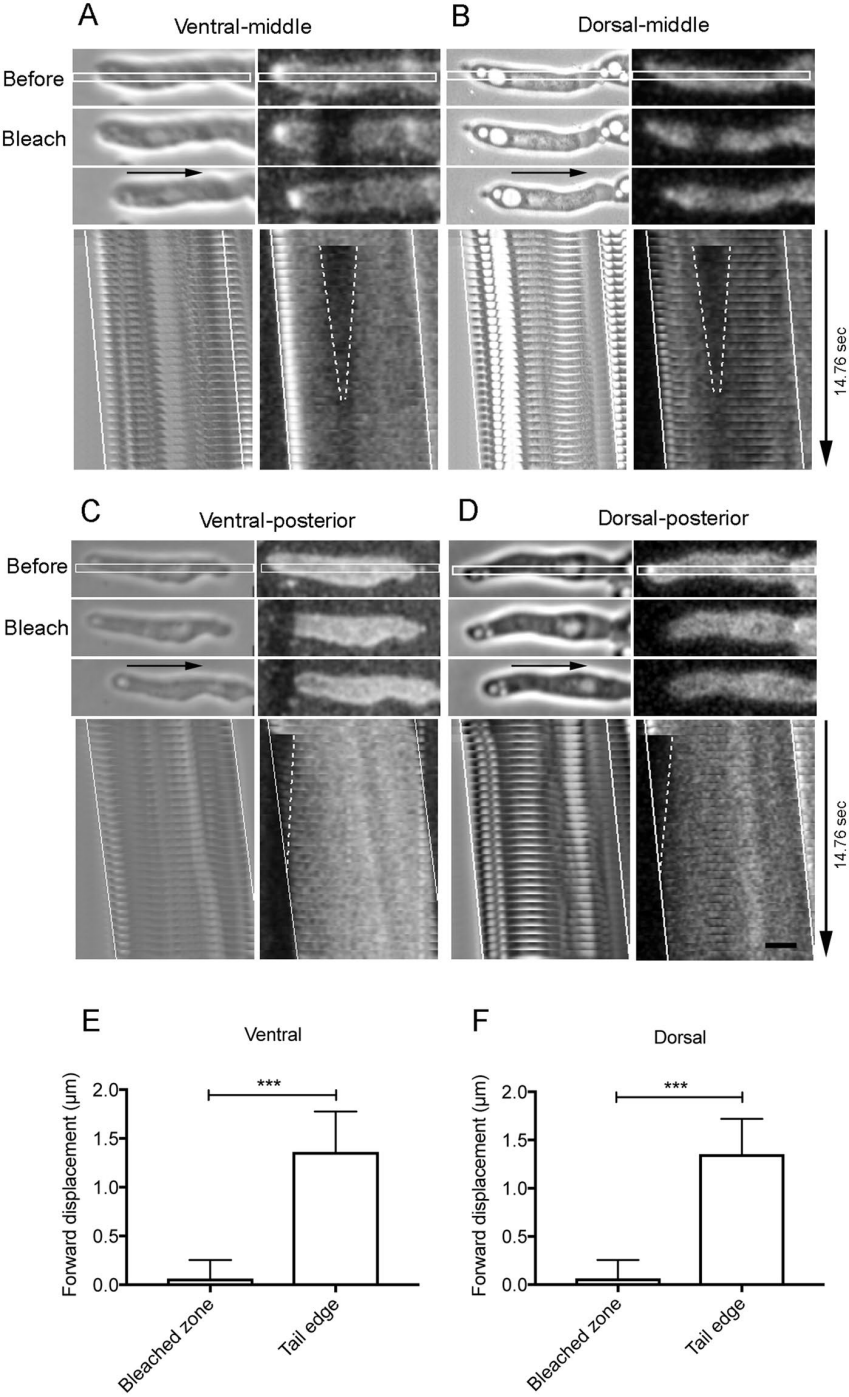
Circulation of the cell membrane during cell migration. Photobleaching experiments in fast-migrating cells. Aggregation-competent cells were stained with Polaric, and the small vertical rectangle regions in the ventral (**A** and **C**) and dorsal cell membrane (**B** and **D**) were photobleached using a confocal microscope. Middle (**A** and **B**) and posterior (**C** and **D**) regions of the cells were bleached, respectively. In each of panel A–D, phase-contrast (left) and fluorescence (right) images before photobleaching, immediately after photobleaching, and after fluorescence recovery are shown. The directions of cell migration are indicated by black arrows. The lower figure in each panel shows a kymograph of the white rectangle shown in each top figure. The white dotted lines show the fluorescence recovery of bleached regions, and the solid lines show the advance of both ends (anterior and posterior) of the migrating cells, respectively. Graphs **E** and **F** show summaries of forward displacements of the bleached zones and tail edges of multiple cells (n = 18 each) in the ventral (**E**) and dorsal (**F**) for 4.8 sec, respectively. Note that the bleached regions did not move relative to the substratum, whereas the cell advanced. Data are presented as mean ± SD and analyzed by unpaired two-tailed Student’s t-test. ***P ≤ 0.0001. Bar, 10 µm.

Figure 6 shows typical results of photobleaching experiments in fast-migrating cells. The dorsal and ventral cell membranes were able to be distinguished by a confocal microscope with the optical depth set to 1 µm (Supplementary Fig. S2). Figure 6A and B show phase-contrast and fluorescence images before photobleaching, immediately after photobleaching, and after fluorescence recovery. Lower panels of Fig. 6A and B show kymographs of the white rectangle in each top figure, which clearly demonstrate that both membranes moved rearward relative to the cells as the cells advanced. Relative to the substratum, the bleached regions did not move.

To investigate whether other membrane regions may behave in a different way from the middle, the anterior and posterior regions were photobleached, respectively. Unfortunately, the anterior region was very sensitive to the photobleaching laser, resulting in stopping cell migration. Figure 6C and D show typical images (phase-contrast and fluorescence) when the posterior regions were photobleached. The kymographs show that both ventral and dorsal membranes moved rearward relative to the cells as the cells advanced, which was similar to the results at middle regions. Figure 6E and F show summaries of forward displacements of the bleached regions and tail edges of multiple cells (n = 18 each) in the ventral (E) and dorsal (F) for 4.8 sec, respectively.

Therefore, the membrane lipids move in a manner consistent with the fountain flow model during cell migration.

## Discussion

In the present study, the total cell surface area did not change, although the cells vigorously changed their shapes with fluctuating velocity during cell migration under agar-overlay. Previous studies have shown a fluctuation of up to 20–30% of the cell surface area in migrating *Dictyostelium* cells^18^. These authors also used agar-overlay to flatten the cells, but the thickness of the cells was approximately 6 µm (the cell diameter is approximately 7–9 µm). In our agar-overlay experiments, the thickness was only 2 µm, which means that cells were much more flattened. Scanning and transmission electron microscopic observations showed that the cell surface had neither noticeable projections nor wrinkles under agar-overlay. This suggests that the cells can migrate without unfolded cell membrane as a membrane reservoir. When the cell membrane is tethered by pulling an attached bead using an optical tweezers, the tension of the membrane has two phases^23^. In the initial phase, the tension does not change even when the tether elongates, suggesting that the membrane reservoir is provided to the cell surface by stretching out membrane folds^23^. By pulling more, in the second phase, the tension abruptly increases, inducing exocytosis from the internal membrane stores. For phagocytosis and cell spreading, a biphasic membrane supply mechanism has been proposed^24,25^. Therefore, we concluded that a reservoir of unfolded membrane is dispensable for cell migration, although we cannot exclude the possibility that it contributes to the cell shape change during cell migration in the absence of agar-overlay. In addition, our data indicate that filopods, which sometimes have been considered to be organelles that help to explore a substratum^26,27^, are dispensable for cell migration.

Previous researchers studied the rate of internalization of the cell membrane in vegetative *Dictyostelium* cells in suspension conditions^12,13,28,29^. Vegetative cells vigorously internalize external nutrient by macropinocytosis, which is not directly related with membrane turnover for cell migration. Thus, here, we measured the internalization of the cell membrane by observing individual migrating cells under non-nutrient conditions. The fastest half-life was approximately 60 s in the present study, which is much faster than previous measurements. This rapid internalization can explain much faster cell migration.

Our photobleaching experiments to evaluate the two models showed that the fountain flow model is more likely to account for the findings described using our system. When a cell is passing through a confined three-dimensional environment such as in a tissue, both lower and upper surfaces of the cell have contact with substratum or surrounding cells. Under this condition, it may be practically difficult to explain the circulation of cell membrane by the caterpillar model. On the other hand, the fountain flow model may be applicable to the circulation of cell membrane in such a confined space.

In the fountain flow model, new cell membrane must be inserted at the anterior of the migrating cell by exocytosis and taken up at the rear by endocytosis. At present, there is no direct observation of such membrane dynamics in *Dictyostelium* cells. However, clathrin, a mediator of endocytosis, localizes at the rear of migrating *Dictyostelium* cells, and clathrin-knockout cells show slow migration, providing evidence that endocytosis mainly occurs in the rear of migrating cells and contributes to cell migration^30^. Clathrin-containing pits and vesicles also mainly appear at the rear of migrating leukocytes and epithelial cells^31,32^. Endocytosis can also occur via a clathrin-independent process, such as a caveolae-mediated process. Caveolin-1-deficient mouse embryonic fibroblasts exhibit poor directionality and non-polarized morphology^33^. Caveolin-1 localizes at the rear of migrating endothelial cells^34^.

Polarized exocytosis at the leading edge for the localized delivery of membranes has been demonstrated in several species of cells. For example, when *Physarum* plasmodium is stained with rhodamine-phosphatidyl ethanolamine, which specifically binds to slime, the slime is found to be exocytosed at the anterior membrane to form protrusions^35,36^. G-glycoprotein synthesized by transfected vesicular stomatitis virus at the Golgi apparatus is transported to the leading edge of migrating fibroblasts, and inhibition of this transport results in defects of directed cell migration^37,38^. The preferential addition of newly synthesized membrane protein occurs at axonal growth cones^39^. Transferrin and low-density lipoprotein receptors are also transferred to the leading edge of carcinoma cells via exocytosis of the internal membrane^40^. Integrins, integral membrane proteins related with cell-substratum adhesion, are recycled to the anterior of migrating neutrophils via a Ca^2+^-dependent mechanism^41^.

Interestingly, previous photobleaching experiments showed that cAR1, a cAMP receptor in the cell membrane, moves forward during cell migration in *Dictyostelium* cells^18^. This observation is inconsistent with the fountain flow model. cAR1 evenly localizes at the cell membrane and is not internalized for a long time^42,43^, which suggests that non-circulating membrane components may behave in a different manner from the circulation of membrane lipids. Whether many circulating membrane proteins behave in a fashion similar to the membrane lipids of *Dictyostelium* cells is unknown at present.

In the present study, to clarify which model is more suitable as a membrane flow model, photobleaching experiments were separately performed on the dorsal and ventral cell membranes in rapidly moving cells. Both photobleached regions did not move relative to the substratum, indicating that the cell membrane moves in a manner consistent with the fountain flow model. Recently, we observed the movement of beads attached to the dorsal cell surface of migrating *Dictyostelium* cells^44^. Interestingly, the beads did not move relative to the substratum as the cell advanced, and the attached beads finally accumulated at the rear of the cells as the cells advanced forward. The cortical actin filaments also did not move relative to the substratum^44^, which is consistent with the present results of the lipid movement (Fig. 6). These pieces of evidence suggest that many cell membrane components, including the cortical actin cytoskeleton, do not move relative to the substratum, supporting retrograde flow.

The fountain flow model assumes that the membrane insertion occurs in the anterior region of migrating cells, and simultaneously, cell membrane uptake occurs in the rear region. Balance between this insertion and uptake generates the retrograde flow of both the dorsal and the ventral cell membrane. Because the total cell area is constant under the agar-overlay condition, the temporal and spatial coordination of exocytosis and endocytosis is essential for cell migration. Cell membrane tension likely regulates such coordination. There are several studies in the literature indicating that an increase in surface tension triggers an increase in exocytosis, and inversely a decrease in surface tension increases endocytosis^45–48^.

How migrating cells maintain a constant cell membrane surface area by coordinating exocytosis and endocytosis and how these membrane dynamics are coordinated with the cytoskeletal systems to drive cell migration remain to be investigated.

## Methods

### Cell Culture

*Dictyostelium discoideum* cells (AX2–214) were cultured in a plastic dish at 22 °C in HL5 medium (1.3% bacteriological peptone, 0.75% yeast extract, 85.5 mM D-glucose, 3.5 mM Na_2_HPO_4_ 12H_2_O, 3.5 mM KH_2_PO_4_, pH 6.3). The extra-chromosomal expression vector GFP-ABD (actin-binding domain of filamin, supplied by Dr. T. Q. P. Uyeda), a probe for actin filaments, was transformed into the cells by electroporation as described previously^49^. The transformed cells were selected in HL5 medium supplemented with 10 μg/ml G418 (Sigma). The cells were washed with BSS (10 mM NaCl, 10 mM KCl, 3 mM CaCl_2_, and 3 mM MES, pH 6.3) and incubated in the same solution for 3–5 h before experiments. In some experiments, to obtain more rapidly migrating cells, the incubation time was extended to 8–10 h, when the cells became aggregation-competent.

### Cell membrane straining

The cells were settled in a glass-bottom plastic dish and 5 µg/ml of CellMask Orange (Invitrogen) was added. A 1 mg/ml stock solution of CellMask Orange was made in dimethyl sulfoxide (DMSO). As a control, a final concentration of 0.5% DMSO did not affect cell migration and morphology. One minute after staining, the excess dye was removed by washing with BSS. The cell membrane was also stained with 0.625 µg/ml of Polaric (PLT-500c6, Goryo Chemical). A 0.25 mg/ml stock solution of Polaric was made in DMSO. In some cases, the cells were overlaid with a 1.5% agar block after staining with these dyes.

### Microscopy

For agar-overlay, a thin agar block (0.17 µm thick) was gently placed on cells attached to coverslips, as described previously^50^. Excess buffer was removed by absorbing with filter paper, until the cells became flat, as assessed by microscopy.

After the agar-overlay, the cells expressing GFP-ABD were observed under an optical sectioning fluorescence microscope (DeltaVision, GE Healthcare Life Science). Six z-axis images with an interval of 0.3–0.5 µm were acquired over time at a time interval of 10 s. Each image was processed by deconvolution to remove out-of-focus fluorescence using a DeltaVision system. Then, the images were processed to make 3D view, and the total cell surface area, cell volume, cell velocity, and circularity were examined using Volocity software (PerkinElmer). Circularity is defined by the following formula (1):

1 $${\rm{Circularity}}=4\pi {{\rm{A}}/{\rm{P}}}^{2},$$

where A is the cell area and P is the cell perimeter, respectively. When the circularity of a cell equals 1, the cell shape is round, and as the shape deviates from a circle, the value decreases.

To observe the cell membrane, cells stained with CellMask Orange were excited with a He/Ne laser (543 nm) and the emission was filtered with a long pass filter (>560 nm). The cells stained with Polaric were excited by an Argon laser (488 nm) and the emission was filtered with a band pass filter (515–530 nm). The cells were observed under a confocal microscope (LSM 510 Meta, Zeiss) equipped with a 100X objective lens (Plan Neofluar, NA 1.3). Time-lapse images were acquired with an interval of 0.1–1 s.

Latrunculin B (Sigma) was dissolved in DMSO to make a 2 mM stock solution. The final concentration of latrunculin B in the experiments was 5 µM in BSS. Thiabendazole (Tokyo Chemical Industry) was dissolved in DMSO to make a 100 mM stock solution. The final concentration of thiabendazole in the experiments was 100 µM in BSS. (−)-blebbistatin (Sigma) was dissolved in DMSO to make a 20 mM stock solution. The final concentration of blebbistatin in the experiments was 150 µM in BSS. Sodium azide (Sigma) was dissolved in distilled water to make a 1.5 M stock solution. The final concentration of sodium azide in the experiments was 15 mM in BSS.

### Photobleaching

Photobleaching was performed using a confocal microscope as previously described^51^. Briefly, the full power of the argon laser (488 nm line) was applied to an assigned region of a cell. The fluorescence intensity in the bleached area was monitored with respect to time after background subtraction. The time course of recovery was fitted to the equation for a single exponential rise to the maximum using ImageJ software (http://rsbweb.nih.gov/ij), and the half-time for recovery was calculated as previously described^51,52^.

To examine the diffusion coefficient, a circular area with 5 µm diameter in the cell membrane was bleached after the cell membrane was stained with Polaric. After calculating the half-life, the diffusion coefficient (D) was calculated by following formula (2):

2 $${\rm{D}}=0.25{{\rm{r}}}^{2}/{{\rm{t}}}_{1/2},$$

where r is the radius of the bleached area and t_1/2_ is the half-life for fluorescence recovery. This formula assumes that the fluorescent probes show a pure 2D diffusional mobility in the cell membrane and photobleaching is performed by a confocal microscope^53^. There was no immobile fraction in the present FRAP experiments.

### Image analysis

The fluorescence intensities of the cell membrane and the cell interior were calculated as follows. The cell shapes were outlined from phase contrast microscopic images. As depicted in Fig. 3G, the integrated fluorescence intensity in a 1 µm-thick outline including the cell membrane was calculated as the fluorescence of the cell membrane. The integrated fluorescence inside the outline was used as the fluorescence of the cell interior. The time course of fluorescence intensity of the cell membrane was fitted to the equation for a single exponential fall to minimum, and that inside the cell was fitted to the equation for a single exponential rise to maximum using ImageJ software.

The mean cell velocity was calculated using ‘manual tracking’ plugin in ImageJ software.

### Scanning electron microscopy

For scanning electron microscopy, cells either with or without agar-overlay were processed by the same method. First, they were fixed by immersing into ethanol containing 1% formalin at −17 °C for 30 min. Then, the solution was exchanged with ethanol/acetone (1:1) for 10 min, and then exchanged with acetone for 10 min. The samples were post-fixed in acetone containing 1% osmium tetroxide on ice for 2 h, and then washed 2 times in absolute acetone for 10 min each. To remove the agar, the absolute acetone was exchanged with ethanol/acetone (1:1) for 10 min, then exchanged with ethanol for 10 min. Then, the ethanol was exchanged with 50% ethanol (diluted with distilled water) and the agar was melted and removed by heating the solution. Then, the solution was exchanged with an ethanol series (50, 75, 95, 100, and 100%) for 20 min each, then exchanged with t-butanol. The samples were finally freeze-dried using a freeze-drier (Eiko, ID-2). In other samples, instead of t-butanol, ethanol was exchanged with HDMS (hexamethyl disilazane) and then the samples were air-dried. After coating with gold using a sputter coater (Sanyu Electron, SC-704), the cells were observed under a scanning electron microscope (JEOL, JSM-6360LA).

### Transmission electron microscopy

The cells on a carbon-coated aluminum sheet of 10 µm thickness with a stainless ring folder were agar-overlaid and quickly frozen in liquid nitrogen-solid nitrogen slush (frozen liquid nitrogen in a vacuum) at −210 °C. The frozen cells were subjected to freeze-substitution with 1% OsO_4_ in acetone at −80 °C for 2 days, dehydrated in absolute acetone at room temperature, substituted with propylene oxide, and then embedded in Spurr’s resin (Sigma-Aldrich). After the resin was cured by a specified method, the aluminum sheet was removed. The cells were serially sectioned at a thickness of 50–60 nm using an ultramicrotome (Leica EM UC7) and stained with 1% uranyl acetate followed by Reynold’s lead citrate^54^. They were observed under electron microscope (Quanta 3D, FEI or CM-120, Philips).

### Statistical analysis

Statistical analysis was performed using GraphPad Prism 7 (GraphPad).

Data are presented as mean ± SD and analyzed by unpaired two-tailed Student’s t-test or one way ANOVA with Tukey’s multiple comparison test.

### Data availability

All relevant data are available from the authors on reasonable request.

## Electronic supplementary material

Supplementary Information

A typical migration of a Dictyostelium cell expressing GFP-ABD under agar-overlay
