# Supplementary material for: Turnover and flow of the cell membrane for cell migration
Source: Sci Rep. 2017 Oct 11;7:12970. doi: 10.1038/s41598-017-13438-5 (PMC5636814; doi:10.1038/s41598-017-13438-5)
Supplement: Supplementary file 1 — Supplementary Information [file 41598_2017_13438_MOESM1_ESM.pdf]

Supplementary Information for

Turnover and flow of the cell membrane for cell migration

Masahito Tanaka, Takeomi Kikuchi, Hiroyuki Uno, and Keisuke Okita, Toshiko Kitanishi-Yumura and Shigehiko Yumura

Supplementary Figure S1

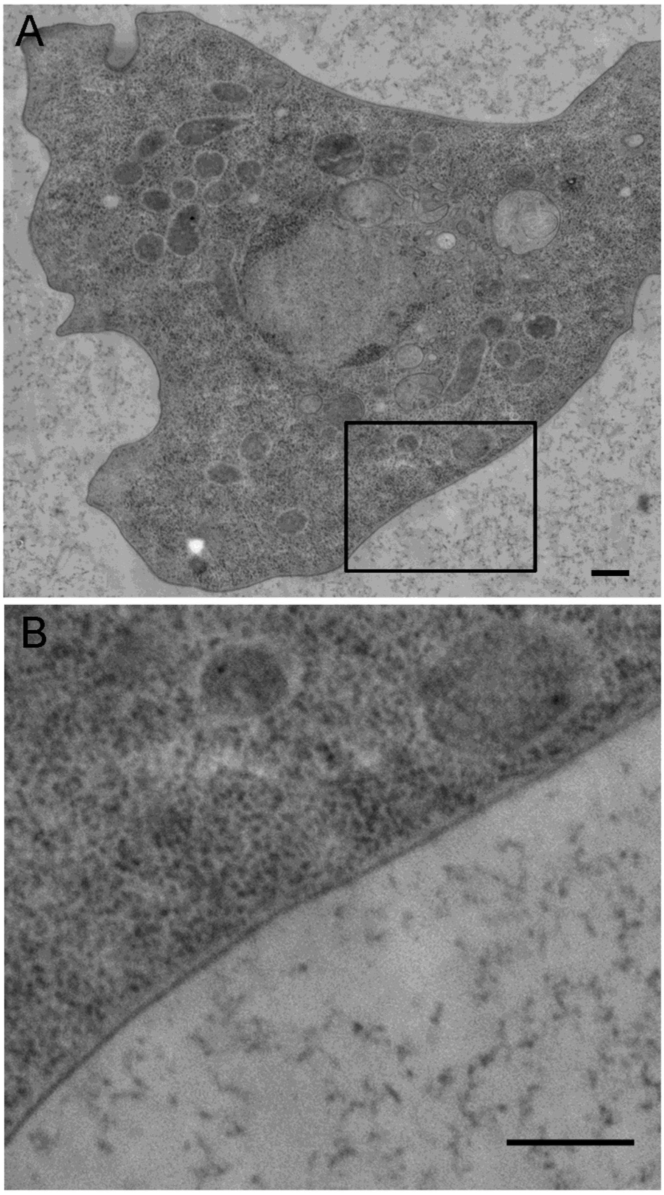

Supplementary Figure S1 Typical transmission electron micrographs of a *Dictyostelium* cell under agar-overlay
Panel B shows an enlarged image of the rectangle in panels A. Under agar-overlay, there was no sign of minute wrinkles or folds on the cell surface. Bar, 1 μm.

Supplementary Figure S2

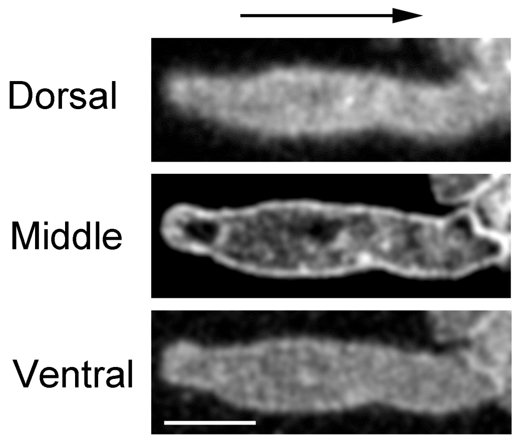

Supplementary Figure S2 Three different optical sections of a *Dictyostelium* cell stained with Polaric

The cell membrane of migrating *Dictyostelium* cells was stained with Polaric. The images were acquired by three z-optical sections (dorsal, middle and ventral) under a confocal microscope. Note that the images were totally different, suggesting that the confocal microscope can distinguish between the dorsal and ventral cell surfaces. Arrow shows the direction of cell migration. Bar, 10 μm.

Supplementary Table S1

Supplementary Table S1 Quantification of cell velocity, surface area and volume in individual migrating cell expressing GFP-ABD under agar-overlay

Cell No.	Mean cell velocity (μm/min)	Maximal changes in relative surface area (%)	Maximal changes in relative volume (%)
1	4.38	6.62	7.51
2	2.81	6.98	7.34
3	3.98	3.87	3.60
4	3.35	4.07	3.64
5	3.44	4.46	5.50
6	2.35	7.56	7.78
7	2.79	4.08	4.02
8	3.59	4.40	5.18
9	2.60	2.56	2.47
10	3.38	3.36	3.84
11	2.25	6.41	6.95
12	4.23	7.64	8.11
13	2.94	2.44	2.64
14	3.71	4.54	3.76
15	2.85	4.63	4.90
16	4.54	7.80	7.70
17	2.91	4.65	4.52
18	4.01	5.33	4.18
19	3.28	4.40	5.18
20	2.66	4.46	4.60
Average	3.30 ± 0.68	5.01 ± 1.62	5.17 ± 1.79

Mean cell velocity, maximal changes in relative surface area and maximal changes in relative volume were measured in 20 different migrating cells. Each cell was observed for at least 10 min under sectioning fluorescence microscopy.

Supplementary Video legend

Supplementary Video S1

A typical migration of a *Dictyostelium* cell expressing GFP-ABD under agar-overlay

The cells expressing GFP-ABD at pre-aggregation stage were agar-overlaid and fluorescence images were acquired at 10 sec intervals under optical sectioning fluorescence microscopy. A z-series of fluorescence images was acquired by optical sectioning fluorescence microscopy, processed using deconvolution to remove out-of-focus signals, and then reconstituted as 3D images. Every time the cell migrated out of the microscopic field, the field of view was changed. The positions of the cell were adjusted in the stacks of the cell boundary shown in Fig 2F.
